# Implementation of A Year-Long Antimicrobial Stewardship Program in A 227-Bed Community Hospital in Southern Italy

**DOI:** 10.3390/ijerph20020996

**Published:** 2023-01-05

**Authors:** Giuseppe Davide Albano, Mauro Midiri, Stefania Zerbo, Emanuele Matteini, Giulia Passavanti, Rosario Curcio, Lidia Curreri, Salvatore Albano, Antonina Argo, Marcello Cadelo

**Affiliations:** 1 Section of Legal Medicine, Department of Health Promotion, Mother and Child Care, Internal Medicine and Medical Specialties, University of Palermo, 90129 Palermo, Italy; 2 Fondazione Istituto G. Giglio, Contrada Pietra PollastraPisciotto, 90015 Cefalù, Italy

**Keywords:** healthcare-acquired infections (HAIs), multidrug-resistant organism (MDRO), antimicrobial stewardship (AMS), patient safety, medico-legal issues, responsibility, litigation

## Abstract

Background: Healthcare-Acquired Infections (HAIs) are serious healthcare complications affecting hospital stay, in-hospital mortality, and costs. Root cause analysis has identified the inappropriate use of antibiotics as the main causative factor in the expansion of multi-drug-resistant organisms (MDRO) in our hospital. An Antimicrobial Stewardship (AMS) program was implemented to optimize antibiotic use, limit the development of resistance, improve therapeutic efficacy and clinical outcomes, and reduce costs. Methods: The stewardship strategies were: antimicrobial oversight on “critical” antibiotics; the development of hospital guidelines on antibiotic selection with the production of a consensus document; the implementation of clinical and management control algorithms with visual impact and Business Intelligence methods; training and updating; and the monitoring of outcome measures and process indicators. Results: Clinical outcomes: length of stay reduced by 0.23 days, hospital readmission/first month rates decreased by 19%, and mortality for infections reduced by 8.8%. Microbiological Outcomes: *Clostridium Difficile* colitis incidence reduced by 9.1%.Economic Outcomes: Reduction in antimicrobial costs by 35% on average fee/discharged patient. Conclusions: The systematic application of the AMS program in a small hospital led to multiple improvements in clinical, microbiological, and economic outcome measures. The analysis of the core indicators for our hospital AMS program showed a significant adherence to the model and hospital recommendations.

## 1. Introduction

Healthcare-Acquired Infections (HAIs) are currently considered the most frequent and severe healthcare complications. They constitute a heavy burden on health systems, affecting hospital days, mortality, and costs [1,2]. Today, out of every 100 patients in acute-care hospitals, 7 in high-income countries and 15 in low- and middle-income countries will acquire at least one HAI during their hospital stay. On average, one in every ten affected patients will die from HAIs [3].

The overuse and inappropriate consumption of antibiotics have driven the emergence and spread of antibiotic-resistant bacteria [4,5]. Over the last several decades, antimicrobial-resistant organisms have been responsible for an increasing percentage of nosocomial infections [6,7,8,9], and infections involving resistant pathogens increase morbidity, mortality, length of hospitalization, and healthcare costs [10,11,12]. Deaths in the world related to antibiotic resistance are estimated at about 4.95 million, including 1.27 million being directly attributable to resistant bacterial strains [13] in 2019.

In a program to control HAIs, cross-sectional studies conducted at the Giglio-Cefalù Hospital-Foundation highlighted a high rate of infections caused by multi-drug-resistant organisms (MDRO). Root cause analysis has identified the inappropriate use of antibiotics as the primary determinant of the expansion of resistant microorganisms.

In this study, we described the organization and operation of a systematic antimicrobial stewardship (AMS) program [14,15,16,17] within the Foundation G. Giglio of Cefalù, a hospital in southern Italy with 227 beds, and its impact on clinical and economic outcomes.

The aim was to improve the treatment of infections through the rationalization of the use of antibiotics in hospitalized patients at the Giglio Foundation with bacterial infectious diseases. We reported the results of one year of application of the program compared with the previous year to demonstrate that our AMS model led to significantly better outcomes and enhanced quality of care. 

We measured the AMS program’s impact through outcome indicators: overall mortality, mortality and intensive care unit (ICU) admission caused by infections, length of stay, and hospital readmission were the clinical outcome measures. The microbiologic outcome measure was the incidence of *Clostridium Difficile* diarrhea. We also evaluated the AMS program’s impact on economic outcomes through antibiotic consumption and cost analysis.

## 2. Materials and Methods

### 2.1. Study Design and Settings

The G. Giglio Foundation, Cefalù, is a community hospital licensed to operate 72 general medical beds, 85 surgical beds, 8 intensive care unit beds, and 62 rehabilitation beds. We conducted a study designed to establish the impact of an AMS program on clinical, microbiological, and economic outcomes by comparison of data collected during the year before and the year after program implementation. The AMS program started on the first of January 2021.The data for the year 2020 were collected retrospectively; for 2021, an information system was developed for the management and monitoring of the activities and to measure the outcome indicators, with dynamic reporting with Business Intelligence systems.

### 2.2. Program Development

Management identified and defined the leadership and accountability of a Working Group (W.G.) for the responsible use of antibiotics. The W.G. acted as a company consultant providing support functions for the AMS in the planning and development phases of actions aimed at optimizing the use of antibiotics and in the annual verification of the results achieved. The W.G. operated as a multidisciplinary team and comprised a panel of operators identified by the infection control team of the hospital based on their expertise in the subject matter and their knowledge of the specific clinical situation in which the model was to be applied.

A coordinator (Referral) was appointed within the W.G. with the function of company referral. The working group included a referral doctor for each Area (Medical, Surgical, Rehabilitation, and Critical Areas), a Referral Doctor for Microbiology, a Pharmacist with specific skills in clinical pharmacology and antibiotic therapy, a Referral Doctor for Risk Management, a Referral Doctor for Legal Medicine Unit and malpractice litigation prevention, a Nurse Specialist in Infectious Risk, and a referral Doctor for Information Systems for the collection and processing of data and the production of reports. The working group was operational from 7 a.m. to 7 p.m. on weekdays and provided night and weekend coverage through smart-working and on-call shifts. It promoted a series of AMS interventions to optimize and standardize antibiotics within the Giglio Foundation. The critical points of the AMS program are summarized in Table 1.

#### 2.2.1. Antimicrobial Oversight

The intervention consists of the supervision by the W.G. Coordinator for the AMS and the Area Referents of the use of antibiotics which, in terms of therapeutic importance, involvement in antibiotic resistance processes, clinical impact, and costs, constitute a “critical” class that therefore requires priority rationalization of their use in therapy (Table 2).

The prescriptive Supervision intervention makes use of two different strategies:

##### “Front End” Strategy

Prescriptive restriction and pre-authorization by the W.G. to prescribe critical antibiotics. Prescribing these antibiotics is restricted, and their use by the unit requires approval by the W.G. team. The pharmacy will have a list of these antibiotics and will only supply the drug after written authorization.

##### “Back End” Strategy

Dissemination of the practice of *audit* and *feedback*, that is, review and verification of the therapies prescribed in the pre-authorization phase through systematic reassessment at 72 h (antimicrobial time-out) and possible adherence to therapeutic de-escalation procedures based on microbiological and clinical parameters. The W.G.’s area contact person or Coordinator reviews and verifies all the therapies prescribed in the pre-authorization phase and practiced at that time in the Foundation in the various hospital units 72 h from the start and provides indications on changes, adjustments, and end of the therapy. The audit and feedback process is documented on a form containing the audit memo, patient data, unit admission, antibiotic treatment implemented, dose, route, frequency of administration, therapeutic indication, and duration of treatment. This procedure is repeated at a timetable set by the W.G. performing the intervention.

Antimicrobial oversight interventions were monitored through specific request forms for antibiotics to the Pharmacy Department, the specific forms to be filled in each antimicrobial timeout performed in “back-end” mode, and through what is documented in the medical record, as suggested in previous studies [18,19,20]. The interventions carried out were proposed by the AMS team whenever a hospital ward requested an AMS suggestion for the authorization to use selected antibiotics that undergo institutional restriction criteria, for a change in antibiotic regimen, or for a consult on microbiologic results. The AMS consult is requested through a specific informatic suggestion form. The AMS team reviewed ongoing treatments after a fixed initial treatment period—the so-called antimicrobial timeout [21,22,23]. The selection of antibiotics subject to monitoring and prescriptive oversight was carried out, taking into account the AWaRe tool developed by the WHO’s Essential Medicines List to tackle antimicrobial resistance, which classifies antibiotics into three groups—Access, Watch, and Reserve—and specifies which antibiotics to choose for the most common infections, which ones are recommended only for specific indications, and which ones should be used only as a last resort [24,25].

#### 2.2.2. Development and Application of Hospital Recommendations and Guidelines

The W.G. has drawn up a hospital consensus document based on the most important scientific evidence on the subject of antibiotic prescription in the hospital setting, contained in the most authoritative and validated International Guidelines, with the main aim of indicating the conditions in which antibiotic therapy is recommended, the type of molecules, the correct dosage, the optimal duration, and the drug administration methods. The document aims to provide up-to-date, evidence-based, and freely available recommendations, adapted to the microbiology and antimicrobial susceptibility patterns present in the Foundation, on the empirical antibiotic treatment of respiratory tract infections; urogenital tract infections; skin and soft tissue infections; gastrointestinal tract infections; ear, nose, and throat Infections; CNS infections; blood infections; and sepsis. Evidence-based guidelines and instruction were diffused among the hospital healthcare workers.

#### 2.2.3. Development and Use of Clinical and Decision Support Algorithms

Implementing clinician choice support systems which have proven effective in reducing treatment errors and improving medical staff compliance with guidelines and protocols was planned. The interventions carried out were developing and using clinical and management control algorithms with visual impact methods and Business Intelligence. The use of apps and tables accessible through computerized therapy was also implemented for adjusting therapeutic dosage according to organ dysfunction (e.g., renal insufficiency) and for optimizing therapeutic dosage according to clinical or pharmacokinetic parameters and interventions guided by the Hospital Pharmacy with alerts in the event of therapeutic anomalies.

#### 2.2.4. Training and Updating

The AMS model of the Giglio Foundation has implemented periodic training and updating activities on the issue, aiming to provide regular updates on antibiotic prescription, antimicrobial resistance, and general management of community- and care-related infections.

### 2.3. Program Outcomes

The goals of the program are to improve prescription appropriateness; limit the development of resistance; enhance therapeutic efficacy and, consequently, patient outcomes; limit the use of antibiotics exclusively to situations where they are indispensable and therefore reduce adverse effects from antibiotics, including those due to over-prescription; and, lastly, cost reduction and a decrease in malpractice litigation related to HAIs. The AMS interventions are summarized in Table 3.

The AMS program provides specific indicators to measure the outcome of the intervention carried out. We distinguished clinical, microbiologic, and economic outcomes.

#### 2.3.1. Clinical Outcomes

− Hospital length of stay;
− Total deaths and deaths from infections;
− Intensive care transfers for infectious complications;
− Unscheduled re-admission to hospital within 30 days of discharge.

#### 2.3.2. Microbiologic Outcomes

− Laboratory diagnosis of *Clostridium Difficile* infection.

#### 2.3.3. Economic Outcomes

− Quantitative consumption of antibiotics;
− Antibiotic cost.

### 2.4. Statistical Analysis

The analysis was performed using R statistical software (R version 4.2.2). Quantitative variables were reported as a mean and standard deviation; the variables included in the analysis do not have a normal distribution; the differences between the analyzed groups (2020 and 2021) were studied with the Wilcoxon test for independent samples. Qualitative variables were reported as absolute terms and percentages, and the differences were evaluated with the Chi-squared test. A *p*-value <0.05 was considered statistically significant. A trend analysis was performed for the significant variables with the Chi-squared test for trend in proportions (i.e., test asymptotically optimal for local alternatives where the log odds vary in proportion with score). Further analysis was conducted by introducing the available data for the year 2022 (January–November).

## 3. Results

### 3.1. Clinical Outcomes

There was a 9.6% reduction in deaths from infection and stable overall mortality (*p* = 0.36).The length of hospital stay was reduced by 0.23 days. The rate of hospital re-admission within the first month from discharge was reduced by 19.1% (*p* = 0.003). The number of ICU admissions from medical and surgical wards for infectious complications reduced by 28% (60/209 vs. 47/228, *p* = 0.04). Microbiological Outcomes: We observed a reduction in the *Clostridium Difficile* stool samples from 16 positives out of 97 in 2020 to 15 out of 171 in 2021, with colitis incidence reduced by 43.4%.

Improvements from previous years were observed from 2022 data (January–November) by comparing them with last years (2020 and 2021). There was a ~12% reduction in overall mortality from the previous year (2021) and a ~19% reduction in deaths from infection over 2021 and a ~26% reduction over the year 2020. The rate of hospital re-admission within the first month after discharge was reduced by ~34% over the year 2020. The ICU admissions from medical and surgical wards for infectious complications reduced by ~43% over the year 2021 and by ~59% over 2020. Microbiological Outcomes: We observed a reduction in the *Clostridium Difficile* stool samples from 16 positives out of 97 in 2020 to 15 out of 171 in 2021, with colitis incidence reduced by 43.4%, and 7 positives out of 101 in 2022,with colitis incidence decreased by 59%.

### 3.2. Economic Outcomes

Quantitative consumption of antibiotics was reduced by 4.8% with a reduction in antimicrobial costs from EUR 432.892 to EUR 332637, with a 23% reduction in the average cost/discharged patient.

The quantitative consumption of antibiotics is monitored by the Pharmacy department. A change in the prescription pattern of many antibiotics has been documented, with a reduction in the consumption of carbapenems, an increase in the consumption of aminoglycosides and semisynthetic penicillins, and a change in the ratio between the glycopeptides used due to an increase in consumption of Vancomycin and a reduction in the use of Teicoplanin and Daptomycin.

**Figure 1:**
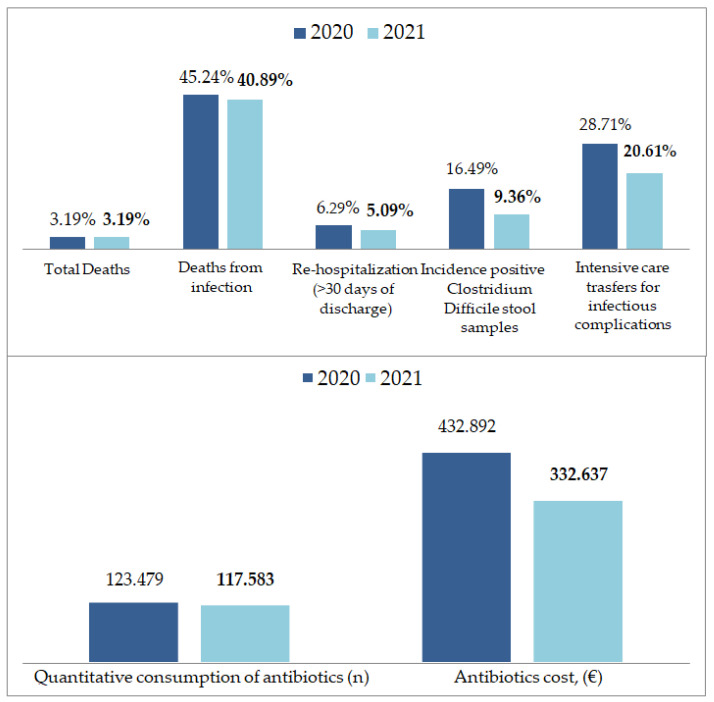
The main AMS program outcome measures and results.

The outcomes of the AMS program and the results are summarized in Table 4 and Figure 1.

Among the clinical outcome measures, total mortality was extrapolated from the analysis of the diagnosis-related-groups (DRGs) that resulted in death and which reported infectious disease in the main or secondary diagnosis and in which the infection had been the cause of or contributed to the patient’s death. The number of admissions to the ICU was obtained from the analysis of medical records that reported at least one ICU admission, excluding all those in which the admission was due to post-operative management or non-infectious complications. The average hospital length of stay data were provided by Management Control, which also provided the cost analysis [26,27]. The number of positive samples for the *Clostridium Difficile* toxin was extrapolated from the active microbiological surveillance data provided by the Microbiology laboratory.

## 4. Discussion

This study describes the application of a comprehensive and systematic antibiotic therapy management program and an AMS program in the G. Giglio Foundation in Cefalù, a 227-bed community hospital, according to the internationally recognized and updated evidence and recommendations in this field. Antimicrobial stewardship is defined as a complex and systematic set of actions aimed at optimizing and standardizing the use of antimicrobial drugs both in community and hospitals through the optimal selection, dosage, route of administration, and duration of treatment to improve patient outcomes, limit the risk of adverse events, and minimize the impact on the development of resistance [21,27,28].

The AMS program of the Giglio Foundation started up according to the recommendations of the Italian Government concerning the fight against HAIs and the National Action plan on Antimicrobial Resistance (PNCAR 2017–2020) [29] and was implemented using only the human and material resources of the Foundation, without any external support. Our model originated from cross-sectional studies performed in the hospital that documented a high number of HAIs caused by MDROs given the well-known relationship between antibiotics consumption and the development of resistance and since their misuse is one of the main factors promoting resistance [30,31]. We largely attributed the spread of MDROs to the inappropriate and unverified use of antibiotics over the past few years in our hospital, as reported in previous studies [32,33]. The primary aim of the Antimicrobial Stewardship model was to ensure the optimal use of antibiotics in patients admitted to the G. Giglio Foundation Hospital with infectious bacterial diseases. We report the results obtained after a year-long application of the program, comparing them with the previous year of activity in which no HAI or AMS control programs were ongoing.

The results obtained showed a trend toward a reduction in total mortality, infections, and the number of ICU admissions. The mortality rate is an important clinical outcome measure, especially in more severe patients, and its reduction can also be an indicator of the safety of the intervention performed [25]. It is less suitable for mild infections and may be affected by other variables, such as deaths from causes unrelated to infections (i.e., STEMI). We extrapolated data on mortality due to infections from overall mortality, with evidence of a more significant reduction. These observations suggest that AMS programs improve efficacy against infections without leading to their undertreatment, as suggested by recent reports [34,35]

We also used the length of stay and rate of re-hospitalization within 30 days of discharge rates as indicators. Although they are sensitive to biases, they have often evaluated intervention measures and have been analyzed in other studies [36]. We observed a statistically significant reduction in these outcome measures partly explained by the high incidence of MDRO and HAIs in the rehabilitation wards in which the hospital stay is longer.

Data on *Clostridium Difficile* incidence, a microbiological indicator strongly related to antibiotic use [37,38], show an increase in the number of samples sent to the laboratory with a proportional percentage reduction in the positive results. These data suggest enhanced awareness of the issue from healthcare workers, leading to more samples being sent to Microbiology whenever they observed diarrhea or Pseudomembranous Colitis suspicion and overall reduced disease incidence.

In addition to the indications provided by the AMS group, the variations in antibiotics consumption and in the prescription pattern are probably also associated to the adoption and application of the Consensus document that contains evidence-based recommendations for the empirical antibiotic therapy of the most common community-acquired infections (CAIs) and HAIs based on the local epidemiology and provides advice for the use of many antibiotics not subject to antimicrobial oversight. Publication of institutional guidelines and education are key steps in developing an AMS program. Various studies highlight that the dissemination and implementation of guidelines, together with the educational processes, increase the effectiveness of AMS programs with favorable effects on antibiotic use, costs, and mortality from multidrug-resistant infections [39].

Although our data need further confirmation given the small size of the sample, many of the results obtained have statistical significance due to the increase in the number of services provided in 2021. The trend appears to be favorable, even where this sign is not reachable. The data indicate an association between the activities of the AMS program and the results obtained, also confirmed by the trend in 2022. Our study offers a detailed description of the key elements and interventions that structure the set of actions of antimicrobial stewardship developed in our hospital, and the data confirm evidence in the literature by suggesting a significant decrease in antimicrobial consumption and cost, although the evidence in the literature does not entirely agree [36,40]. We believe that the AMS programs improve the treatment of infections and can reduce antimicrobial resistance and the risk of HAIs. Moreover, AMS program efficacy may impact the prevention of malpractice liability and the compensation costs linked to HAI-related litigation, which is considered a significant economic burden for the Health System [41,42,43,44]. Furthermore, the AMS program leads to substantial benefits in terms of quality of care and patient safety, representing a key element in ensuring higher standards of care.

## 5. Conclusions

The proposed AMS program led to significant clinical and economic outcomes in our hospital after a year. A reduction in antibiotics consumption and higher adhesion to evidence- and guidelines-based clinical practices were observed among healthcare workers, with a considerable decrease in costs and an increase in quality of care. To ensure clinical governance and patient safety goals and to decrease medical malpractice litigation related to HAIs, AMS programs should be promoted and implemented in all healthcare settings.

A one-size-fits-all stewardship program is not suitable because different groups of professionals have various skills, and healthcare institutions have different rules and organization issues and provide different services. Furthermore, the evidence on the effects of antimicrobial stewardship models is not always conclusive, and outcome measures can be sensitive to biases.

The model we described was effective for a medium-sized hospital and the indicators measured show an association with an improvement in the quality of care.

## Figures and Tables

**Table 1:** A summary of the antimicrobial stewardship program’s key points (HAIs: hospital-acquired infections; AMS: antimicrobial stewardship; CAIs: community-acquired infections).

Organization	Our Institution Developed an AMS Program
Education	Promotion of education campaign for all healthcare personnel about HAI prevention and control and AMS.
Good Clinical Practices and Guidelines	Sharing good clinical practices and evidence-based guidelines in infection control and AMS among healthcare personnel.
Antimicrobial Stewardship Program	Antibiotic prescription restriction and supervision; clinical choice support for antimicrobic therapy; training and updating; higher standard of care in managing CAIs and HAIs; availability of AMS team in the hospital.
Audit and Feedback	Dissemination of practice of audit and feedback about prescription and authorization of therapeutic plans.
Surveillance	Medical records and pharmacy activity in-depth evaluation regarding antibiotic prescription and use.
Results Monitoring	Comparison of results after one year of AMS program.

**Table 2:** Antibiotics used with Antimicrobial Stewardship oversight.

Carbapenems	Imipenem-Cilastatin, Meropenem, Ertapenem, Doripenem
Carbapenems + β-lactamase inhibitor combination	Meropenem/Vaborbactam, Imipenem/Relebactam
Oxazolidinones	Linezolid, Tedizolid
Lypo(Glico)peptides	Daptomicin, Dalbavancin, Oritavancin
Streptogramin	Fosfomycin
Polymyxin	Colistin
β-lactam/β-lactamase combination	Ceftazidime-Avibactam, Ceftolozane-Tazobactam

**Table 3:** The AMS interventions performed.

Interventions
Authorization for restricted antibiotics
Conversion of intravenous to oral for high-bioavailability antibiotics
Switch from broad spectrum to narrow spectrum
Descalation therapy based on microbiologic data
Stop antibiotics if no infection is diagnosed
Stop antibiotics if criteria to define healing are present
Step-down therapy
OPAT

**Table 4:** The AMS program outcome measures and results.

	2020	2021	2020 vs. 2021	*p*-Value
Admissions/year, *n* (%)	6582 (31.72%)	7053 (33.99%)	0.0714	<0.001
Total deaths, *n* (%)	210 (3.19%)	225 (3.19%)	−0.01%	0.2073
Deaths from infection, *n* (%)	95 (45.24%)	92 (40.89%)	−9.62%	0.3599
Hospital stay, mean (SD)	10.8 (20.5)	10.3 (18.9)	−4.06%	0.0324
Re-hospitalization (>30 days of discharge), *n* (%)	414 (6.29%)	359 (5.09%)	−19.08%	0.0025
Quantitative consumption of antibiotics, (*n*)	123.479	117.583	−4.77%	<0.001
Incidence positive *Clostridium Difficile* stoolsamples, *n* (%)	16 (16.49%)	16 (9.36%)	−43.24%	0.0833
Intensive care transfers, *n* (%)	209 (3.18%)	228 (3.23%)	1.57%	0.1958
Intensive care transfers for infectious complications, *n* (%)	60 (28.71%)	47 (20.61%)	−28.21%	0.0493
Antibioticscost, (EUR)	432.892	332.637	−23.16%	<0.001

## Data Availability

All the data are available by request to the corresponding author.
